# Influence of ylHog1 MAPK kinase on *Yarrowia lipolytica* stress response and erythritol production

**DOI:** 10.1038/s41598-018-33168-6

**Published:** 2018-10-03

**Authors:** Dorota A. Rzechonek, Alison M. Day, Janet Quinn, Aleksandra M. Mirończuk

**Affiliations:** 1 Wrocław University of Environmental and Life Sciences, Department Of Biotechnology and Food Microbiology, Chełmońskiego 37, 51-630 Wrocław, Poland; 2 Newcastle University, Institute for Cell and Molecular Biosciences, Framlington Place, Newcastle upon Tyne, NE2 4HH United Kingdom

## Abstract

Erythritol production is a unique response to hyperosmotic stress that is observed in a small group of yeasts, including *Yarrowia lipolytica*. This study investigated whether this unusual mechanism is regulated by the HOG pathway, well described in *Saccharomyces cerevisiae*. The gene *YALI0E25135g* was identified as the *Y. lipolytica* homologue of *HOG1* and was found to be phosphorylated in response to hyperosmotic shock. Deletion of the gene caused a significant decrease in resistance to hyperosmotic stress and negatively affected erythritol production. Interestingly, the deletion strain *yl-hog1Δ* displayed significant morphological defects, with the cells growing in a filamentous form. Moreover, *yl-hog1Δ* cells were also resistant to the cell wall damaging agents Congo red and calcofluor white. Collectively, these results indicate that yl-Hog1 is crucial for the cellular response to hyperosmotic stress, plays a role in the induction of erythritol production, and potentially prevents cross-talk with different MAPK signalling pathways in the cell.

## Introduction

*Yarrowia lipolytica* is an unconventional, dimorphic yeast with great potential for industrial applications. It is a good producer of value-added compounds such as lipids, polyols and organic acids^1,2^. In recent years, a number of metabolic pathways have been identified and *Y. lipolytica* has been subjected to numerous genetic modifications in order to optimize the production of compounds such as functional fatty acids, carotenoids^3^ or erythritol^4^. Considering the high interest in this yeast for industrial purposes, it is surprising that very little is known about the regulation of metabolic and signalling pathways. This lack of knowledge was recognized during attempts to optimize erythritol production^5,6^. This four-carbon polyol can be used as an almost zero-calorie sweetener in the food industry and is produced by *Y. lipolytica* from glucose or glycerol^4^. The efficiency of erythritol production is dependent on the high osmotic pressure of the culture medium, and thus media of high osmolarity are commonly used^5,7–10^. However, the mechanism by which osmotic stress stimulates the production of this polyol is unknown.

**Figure 1:**
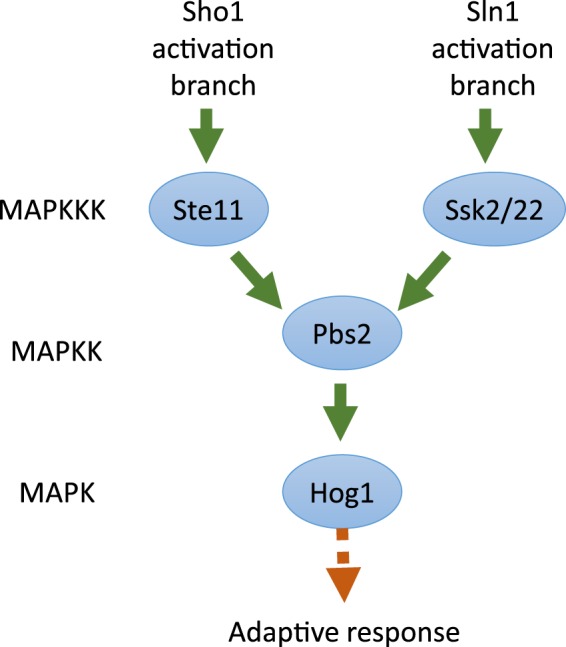
MAPK pathway leading to activation of Hog1 in *S. cerevisiae*.

Rapid cellular responses to stressful conditions is essential for survival in changing environments. Mitogen activated protein kinase (MAPK) pathways, which sense and respond to stressful environments, are found in all eukaryotic organisms^11^. Probably the best described MAPK pathway is the hyperosmotic stress-sensing high osmolality glycerol (HOG) pathway in the model yeast *Saccharomyces cerevisiae*^12–14^. The key element is the terminal MAPK, Hog1, which is activated following the dual phosphorylation of conserved threonine and tyrosine. Phosphorylation of Hog1 is carried out by the upstream Pbs2 MAPK kinase (MAPKK), which in turn is activated by phosphorylation which is mediated by one of three MAPKK kinases (MAPKKKs): Ste11, Ssk2 or Ssk22 (Fig. 1). These MAPKKKs are regulated, in turn, by two distinct upstream osmosensing signalling branches. The Ssk2/Ssk22 MAPKKKs are regulated by the Sln1 branch, while the Ste11 MAPKKK functions downstream of the Sho1 branch of the HOG pathway (reviewed in^13^).

Following phosphorylation, the Hog1 MAPK induces a multi-faceted response to osmotic stress which includes the induction of stress-protective genes, transient cell cycle arrest, and the synthesis and retention of the osmoprotectant glycerol^12,13^. Key to promoting survival following acute osmotic stress appears to be the rapid increase in intracellular levels of glycerol. Increases in glycerol levels are accomplished by the Hog1-mediated post-transcriptional regulation of glycolytic and glycerol biosynthesis genes and the induction of glycerol biosynthesis genes, and the Hog1-mediated prevention of glycerol efflux via closure of the Fps1 glycerol channel^12,13^.

Similar Hog1 MAPK pathways have been described in other yeasts and also filamentous fungi^15^. Hog1 itself is highly conserved; however, there is evidence of divergence in both the regulatory elements and the nature of the stress-signals relayed to Hog1 across the fungal kingdom. For example, in *Candida albicans*, the Sho1-Ste11 branch does not relay osmotic stress signals to Hog1. This is mediated solely by the Ssk2 MAPKKK, which is regulated by both Sln1 dependent and independent mechanisms^16^, while the extremely halotolerant *Hortaea werneckii* has two redundant Hog1 homologues^17^. Moreover, in the model yeast *Schizosaccharomyces pombe*, there is no Sln1 homologue and instead distinct histidine kinases relay oxidative and not osmotic stress signals to the Hog1 homologue Sty1/Spc1^18^.

In addition to osmotic stress, subsequent studies have indicated that the HOG pathway in *S. cerevisiae* also responds to stresses such as oxidizing agents, heavy metals, weak acids, and heat or cold shock^14^. Hog1 also plays vital roles in preventing cross-talk to other MAPK pathways. For example, in the absence of a functional Hog1 pathway osmotic stress triggers inappropriate activation of the Fus3 and Kss1 MAPKs which regulate filamentous growth, cell wall integrity, and mating^12^. Thus some phenotypes observed in *S. cerevisiae hog1Δ* mutants are due to the inappropriate activation of alternative MAPKs.

Interesting, the adaptive response of *Y. lipolytica* to hyperosmotic stress is very different to that well characterized in *S. cerevisiae*. Glycerol does not seem to accumulate inside cells; instead it is used as a substrate to produce erythritol. Moreover, even though the intracellular concentration of erythritol increases several fold under hyperosmotic conditions, a significant fraction of this polyol is released into the growth medium^19^. This brings into question whether such responses are regulated by the HOG pathway. Thus, the aim of this study was to investigate the role of Hog1 in regulating *Y. lipolytica* stress responses and its potential involvement in erythritol production.

## Materials and Methods

### Strains and culture conditions

Strains used in this study were *Y. lipolytica* MK1^20^ and AMM, which is a *∆ura3* derivative of MK1^21^. These strains were obtained from the Department of Biotechnology and Food Microbiology at Wroclaw University of Environmental and Life Sciences, Poland. The strains used in the current study are listed in Table 1.

**Table 1:** Strains used in the study.

Strain	Genotype or plasmid	Source
***E. coli***		
DH5α		Laboratory strain
DH5α	pQE80	Qiagen
DH5α	pAD, UAS1_B16_TEF promoter	^25^
DH5α	JM1133	^24^
DH5α	pQE80-ptHog1	This study
DH5α	pQE80-ptHog1Ura	This study
DH5α	pAD-HOG1	This study
***Y. lipolytica***		
MK1	*MATA*	^20^
AMM	*MATA*, ura3-302	^21^
AMM *yl-hog1∆*	*MATA*, ura3-302, *hog1*::*URA3*	This study
AMM *yl-HOG1*	*MATA*, ura3-302, pAD-HOG1::*URA3*	This study
AMM *yl-hog1∆* ura^−^	*MATA*, ura3-302, *hog1*	This study
AMM *yl-hog1∆* yl-HOG1	*MATA*, ura3-302, *hog1*, pAD- HOG1::*URA3*	This study

*Escherichia coli* strains, used in the transformation procedures, were cultivated in LB medium according to standard protocols^22^. Sterile liquid YNB medium without amino acids (Sigma-Aldrich) supplied with 2% (w/v) glucose was used for yeast transformation.

Sterile yeast extract peptone glucose (YPD) medium, containing 1% (w/v) yeast extract (Merck, Germany), 1% (w/v) peptone (Merck) and 2% (w/v) glucose, was used to obtain yeast biomass for DNA extraction and inoculum preparation. Liquid YPD media supplemented with different concentrations of NaCl were used in Bioscreen C and shake-flask experiments. Shake-flask experiments were performed in 0.3 L Erlenmeyer flasks containing 0.05 L medium on a rotary shaker (CERTOMAT IS, Sartorius Stedim Biotech) at 30 °C and 240 rpm.

Erythritol synthesis was conducted in Erythritol Synthesis Medium (ESM medium) containing 100 g/L glycerol (Chempur, Poland), 2.3 g/L (NH_4_)_2_SO_4_ (Chempur), 1 g/L MgSO_4_ x 7H_2_O (Chempur), 0.23 g/L KH_2_PO_4_ (Chempur), 26.4 g/L NaCl (Chempur), 1 g/L yeast extract and 3 g/L CaCO_3_, pH 3.0. CaCO_3_ was added after establishing pH 3 in order to prevent a drop in pH during culture. Erythritol synthesis was performed in the shake-flask experiments in 0.3 L flasks with baffles containing 0.05 L of medium kept on a rotary shaker at 28 °C and 240 rpm.

### Analysis of conserved sequences

The sequence of the gene encoding *Y. lipolytica* Hog1 was obtained by comparison of the *Y. lipolytica* CLIB122 genome^23^ with *S. cerevisiae* and *C. albicans HOG1* sequences using the BLASTp algorithm on the National Centre of Biotechnology Information (NCBI) website. The gene sequence *YALI0E25135g* and flanking regions were used to design primers to allow for its deletion and overexpression (listed in Table 2).

**Table 2:** PCR primers used in the study.

Primer	Sequence (5′ → 3′)	Purpose
PF-HindIII	CGGAAGCTTGGTCTATGATGCTGCAAGTC	yl-Hog1 deletion
PR-I-SceI	CTTATTACCCTGTTATCCCTATAAAGTCCGCCATGTTACG	yl-Hog1 deletion
TF-I-SceI	CAATAGGGATAACAGGGTAATCCAAACAATGTGGGTCAAAG	yl-Hog1 deletion
TR-NheI	CGGGCTAGCAGAATGGCGTGTTGAATATG	yl-Hog1 deletion
ptHog1-col-F	TTTGGTGCAGCAAGCACAAG	Evaluation of deletion
ptHog1-col-R	GATGGCGTGTCATCACATCG	Evaluation of deletion
Hog1-AscI-F	AATGGCGCGCCTAACATGGCGGACTTTATC	yl-Hog1 overexpression
Hog1-NheI-R	AGAGCTAGCACATTGTTTGGGTGTTTACTG	yl-Hog1 overexpression
col-Hog1-F	CTGAAGTACGTGCACTCTG	Evaluation of overexpression
col-Hog1-R	CTCGGTGATGATGGAGAAC	Evaluation of overexpression
TEF-seq-F	GTCAACTCACACCCGAAATC	Evaluation of overexpression

### Construction of *yl-Hog1* deletion cassette

The promoter and terminator regions of the gene *YALI0E25135g* were amplified by PCR using the primers PF-HindIII/PR-I-SceI for the promoter region and TF-I-SceI/TR-NheI for the terminator region. The PCR reactions were performed using Phusion high-fidelity DNA polymerase (Thermo Scientific) with *Y. lipolytica* MK1 genome DNA as a template. The amplified PCR promoter fragment (1068 bp) was digested with *HindIII* and *I-SceI*. The PCR terminator fragment (1008 bp) was digested with *NheI* and *I-SceI*. Both fragments were cloned into the plasmid pQE80 (Qiagen), digested with *NheI* and *HindIII*. The resulting plasmid, pQE80-ptHog1, was digested with *I-SceI* and a lox1-Ura-lox4 fragment (excised from the plasmid JM1133)^24^ was ligated into the *I-SceI* site. This resulted in the creation of the plasmid pQE80-ptHog1Ura, which carries the ylHog1-Ura disruption cassette. Successful plasmid creation was validated by PCR with the primers ptHog1-col-F and ptHog1-col-R.

### Construction of *yl-Hog1* overexpression cassette

The gene *YALI0E25135g* was amplified from *Y. lipolytica* genomic DNA with Phusion polymerase and primers Hog1-AscI-F and Hog1-NheI-R, resulting in a 1200 bp PCR fragment. The fragment was digested with *AscI* and *NheI* (FastDigest Thermo Scientific) and cloned into the corresponding sites within the plasmid pAD^25^, carrying the UAS1B_16_-TEF promoter^26^. This resulted in the production of the plasmid pAD-HOG1, which drives the expression of *ylHog1* from the *TEF* promoter. Successful plasmid creation was validated by two PCR reactions with the primers TEF-seq-R/col-Hog1-R and col-Hog1-F/col-Hog1-R.

### Cloning and transformation methods

Transformations of *Yarrowia lipolytica* were performed using the lithium acetate method^27^. The deletion cassette was excised from the plasmid pQE80-ptHog1Ura using the enzymes *HindIII* and *NheI* (Thermo Fisher Scientific). The cassette was transformed into the *Y. lipolytica* strain AMM^21^, which is an MK1 derivative auxotrophic for uracil. Ura + transformants were analyzed for proper integration of the cassette by genomic DNA extraction and PCR validation using primers ptHog1-col-F and ptHog1-R. The resulting strain was named AMM *yl-hog1∆*. Uracil auxotrophy of AMM *yl-hog1∆* cells was restored by the Cre-lox recombinase system following transformation of the replicative plasmid pUB4-Cre1(JME547)^24^. This resulted in the strain AMM *yl-hog1∆* ura-.

The overexpression cassette was excised from the plasmid pAD-HOG1 using the enzyme *MssI*. The copy of the *YALI0E25135g* gene and the UAS1B_16_-TEF promoter were surrounded by *Y. lipolytica* rDNA for targeted integrations. The cassette was used to transform *Y. lipolytica* AMM or *Y. lipolytica* AMM *yl-hog1∆* ura- cells, resulting in the strains AMM *yl-HOG1* and AMM *yl-hog1∆* yl-*HOG1*, respectively. Correct integration was verified by gDNA extraction and PCR analysis with the primers col-Hog1-F and col-Hog1-R.

### Stress sensitivity tests

*Y. lipolytica* strains were grown to the exponential phase (OD_600_ = 0.6) in liquid YPD medium at 30 °C. 1, 10^−1^, 10^−2^ and 10^−3^ dilutions were spotted on sterile YPD agar plates, containing the chosen stress-inducing agents. Plates were incubated at 30 °C for 48 h, with the exception of cultures tested for sensitivity for higher or lower temperatures (range from 4 to 37 °C, plates at 4 °C were incubated for 7 days). Additives to the YPD plates were: H_2_O_2_ (1–5 mM), menadione (0.1–0.4 mM), NaCl (0.05–1 M), sorbitol (0.05–1 M), Congo red (10–200 µg/ml), Calcofluor white (0.1–10 µg/ml).

Sensitivity to NaCl in liquid medium was also tested using the Bioscreen C system (Oy Growth Curves Ab Ltd., Finland). The yeast strains were grown in 100-well plates in 150 μL of YPD supplemented with NaCl (0–5%). The cells were inoculated to an OD_600_ value of 0.15 in each well. Quintuple experiments were performed at 30 °C under constant agitation and growth was monitored by measuring the optical density at 420–560 nm every 30 min for 72 h.

### Western blotting analysis

Yeast strains were grown in YPD medium to the mid-exponential phase (OD_660_ = 0.6) and exposed to stress for 10 and 20 minutes. 20 mL of culture was harvested at each step and immediately frozen by immersion in liquid nitrogen. Protein extraction was performed in 0.2 M Tris-HCl buffer pH 7.5 with IGEPAL CA-630 (0.5%), imidazole (10 mM), protease inhibitors (leupeptin, pepstatin A, aprotinin, PMSF) and phosphatase inhibitors (sodium vanadate, sodium fluoride, β-mercaptoethanol) and cells were broken with Mini-BeadBeater-24 as described previously^28^. Protein extracts (40 µg) were resolved by SDS-polyacrylamide gel electrophoresis on 10% gels and transferred to nitrocellulose blotting membrane (GE Healthcare). PageRuler Prestained Protein Ladder (10 to 180 kDa) was used as a marker. The detection of phosphorylated Hog1 using an anti-phospho-p38 antibody (New England Biolabs) and total levels of Hog1 using an anti-Hog1 antibody (Santa Cruz Biotechnology) was performed as described previously^16^.

### Microscopy

Differential interference contrast (DIC) images of yeast cells were captured using a Zeiss Scope A1 Microscope using a Plan-Neofluar 40x objective. Images were taken of the indicated strains following 24 and 48 h growth in liquid YPD supplemented with 0, 0.2 and 0.9 M NaCl concentration.

### Analytical methods

The concentrations of polyols and organic acids were determined by HPLC using a HyperRez Carbohydrate H+ Column (Thermo Scientific, Waltham, MA) coupled to a UV (*λ* = 210 nm) (Dionex, Sunnyvale, USA) and a refractive index detector (Shodex, Ogimachi, Japan). 0.25% trifluoroacetic acid was used as a mobile phase solvent. The samples were diluted 10-fold before the measurement. Data were analysed with the Chromeleon program.

## Results

Based on BLAST comparisons with *S. cerevisiae* and *C. albicans HOG1* sequences, the gene *YALI0E25135g* was identified as the *Y. lipolytica* homologue of Hog1. It encodes a protein of 386 amino acids, which is shorter than *S. cerevisiae* Hog1 (435 aa), and slightly longer than *C. albicans* Hog1 (377 aa). The identity to *S. cerevisiae* Hog1 is 85% and to *C. albicans* 80%. Thus *YALI0E25135g* is referred to as yl-Hog1 for the remainder of the manuscript.

*Y. lipolytica* strains in which yl-Hog1 was deleted (*yl-hog1∆*) or over-expressed (*yl-HOG1*) were created. In addition, a complemented *yl-hog1∆* deletion strain was created by introducing the overexpression cassette (strain *yl-hog1∆*/*yl-HOG1*). Complementation revoked the effects of yl-Hog1 deletion, but the expression of yl-Hog1 in *yl-hog1∆*/*yl-HOG1* cells from the strong UAS1_B16_TEF promoter^26^ resulted in phenotypes more similar to the overexpression strain *yl-HOG1* than wild MK1.

### Response to osmotic stress

**Figure 2:**
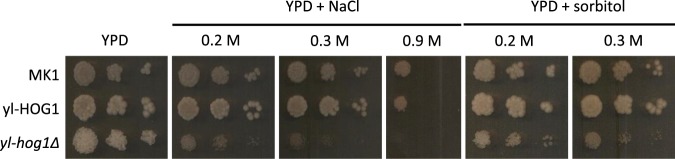
Growth of *Y. lipolytica* strains MK1, *yl-hog1Δ* and yl-HOG1 after 48 h on YPD agar medium supplemented with different concentrations of NaCl or sorbitol.

As the HOG pathway is universally required for osmotic stress resistance in fungi, we initially examined the effect of deleting or overexpressing ylHog1 on the resistance of *Y. lipolytica* to osmotic stress. Exponentially growing cells were spotted onto YPD agar plates containing NaCl (Fig. 2). After 48 hours of incubation on control YPD plates (no stress), the strains MK1 (wild-type), yl-HOG1 and yl-hog1∆ all displayed similar growth characteristics, although, interestingly, the morphology of *yl-hog1∆* colonies was notably different. In contrast, the growth of *yl-hog1∆* cells was considerably impaired in the presence of all concentrations of NaCl tested, compared to MK1 wild-type cells. Overexpression of yl-Hog1 in the strain *yl-HOG1* did not however increase resistance to NaCl stress compared to wild type MK1 cells. This is likely related to the fact that such kinases are regulated at the post-transcriptional level by phosphorylation. The results for the strain *yl-hog1∆*/*yl-HOG1* were omitted due to their similarity to *yl-HOG1*. To ensure that the observed phenotypes were the result of osmotic pressure, and not cationic stress caused by NaCl, sorbitol was also used to increase the osmolality of the medium. As seen with NaCl, *yl-hog1∆* cells were clearly more sensitive to sorbitol than wild-type cells. Thus ylHog1 is needed for osmotic stress resistance in *Y. lipolytica*.

**Figure 3:**
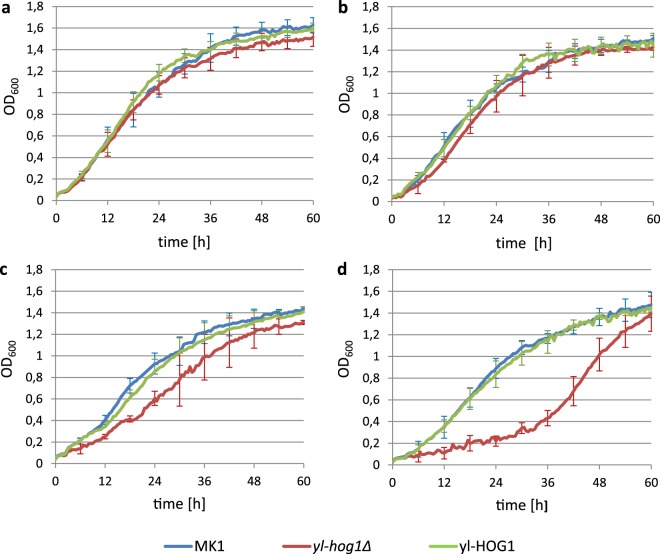
Growth of *Y. lipolytica* strains MK1, *yl-hog1Δ* and *yl-HOG1*, in YPD medium supplemented with different concentrations of NaCl: (**a**) without NaCl, (**b**) 0.2 M NaCl, (**c**) 0.4 M NaCl, (**d**) 0.9 M NaCl. OD_600_ changes were measured by Bioscreen C. Average was counted from eight repetitions.

The effect of NaCl-induced osmotic stress on cell growth in liquid culture was also tested. The growth rate of *Y. lipolytica* cells was evaluated by changes in OD measured using the Bioscreen C system. Under liquid growth conditions, all strains were less sensitive to changes in osmotic pressure compared to the sensitivity seen in solid media, and a higher concentration of NaCl was necessary to detect impaired growth of *yl-hog1Δ* cells (Fig. 3). In the absence, or low concentration (0.2 M), of NaCl in the medium, the growth pattern of all examined strains was similar (Fig. 3a,b). A slightly impaired growth rate of *yl-hog1∆* cells was observed in the presence of 0.4 M NaCl (Fig. 3c). However, 0.9 M NaCl had a dramatic effect, inducing an approximate 36 h lag in the growth in *yl-hog1∆* cells, which was followed by rapid growth (Fig. 3d). Whilst an increase in osmotic pressure (0.4 M and 0.9 M NaCl) also slowed down the growth rate of strains expressing yl-Hog1, there was no long lag phase. Moreover, similar to the results with the spot tests, overexpression of yl-Hog1 in the strain *yl-HOG1* did not increase the growth rate upon exposure to high NaCl concentrations compared to the wild-type MK1 strain.

### Role of yl-Hog1 in promoting resistance to additional stress conditions

**Figure 4:**
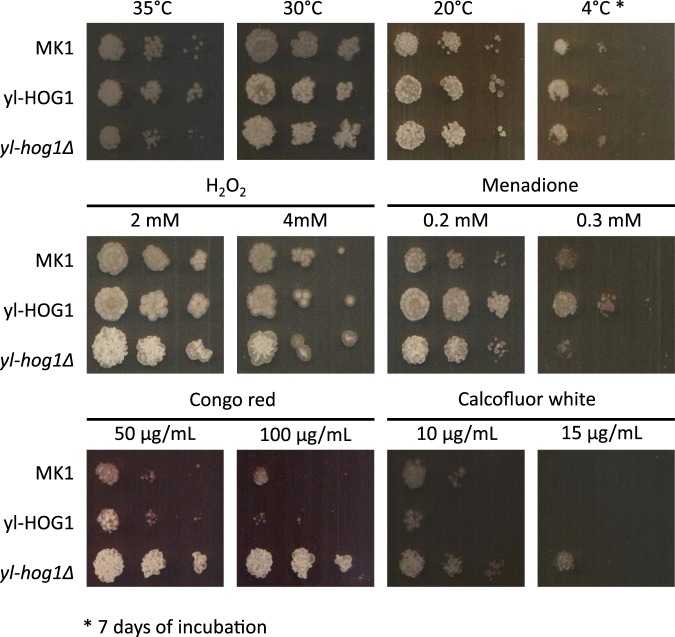
Growth of *Y. lipolytica* strains MK1, *yl-hog1Δ* and yl-HOG1 after 48 h on YPD agar medium either at the indicated temperature, or supplemented with the indicated stress agents.

Studies on Hog1 MAPK cascades from other yeast species have shown that Hog1 is important for cellular survival in the presence of a wide range of stresses in addition to high osmolality. To explore addition stress protective roles for yl-Hog1 in *Y. lipolytica*, MK1, *yl-hog1∆* and yl-*HOG1* strains were tested for resistance to different temperatures, oxidative stress, and factors disturbing the cell wall (Fig. 4). Such conditions were chosen based on the role of Hog1 homologues in promoting resistance to such stresses in *S. cerevisiae*, *C. albicans* and *S. pombe*^14^. If not indicated otherwise, YPD plates were incubated at 30 °C for 48 h.

First, the role of yl-Hog1 in promoting growth at different temperatures was tested (Fig. 4 upper panel). The optimal temperature for *Y. lipolytica* growth and biotechnology purposes is 28–30 °C, but it can also grow at lower temperatures. During incubation at 30 °C and 20 °C all strains displayed similar growth. Growth of *Y. lipolytica* was also observed at 4 °C after extended incubation for 7 days. However, even under these conditions, there was no impact of loss of *yl-hog1∆* on growth. At higher temperatures of 35 °C, more colonies of *yl-HOG1* survived compared to MK1, while colonies of *yl-hog1∆* were slightly smaller than other strains. It was hoped that the difference would be more pronounced at the higher temperature of 37 °C, but this temperature proved to be lethal for all strains (data not shown).

To investigate the role of yl-Hog1 in oxidative stress resistance, the same panel of strains was spotted onto plates containing either H_2_O_2_ or the superoxide generator menadione (Fig. 4 middle panel). Interestingly, the addition of H_2_O_2_ resulted in larger but fewer colonies. However, the strain *yl-hog1∆* was not notably more sensitive than the wild-type MK1 strain in the presence of either menadione or H_2_O_2_. Interestingly, however, overexpression of yl-Hog1 in the strain *yl-HOG1* resulted in higher resistance to menadione (0.3 mM), but not H_2_O_2_. Taken together, however, these data indicate that yl-Hog1 is dispensable for wild-type levels of resistance to oxidative stress.

In both *S. cerevisiae*^29^ and *C. albicans*^30^, *hog1Δ* cells are more resistant to cell wall damaging agents due to crosstalk resulting in the inappropriate activation of the Kss1/Cek1 cell wall integrity MAPK pathways. To explore the impact of yl-Hog1 loss on resistance to cell wall damaging agents, the same panel of strains as above were spotted onto plates containing either Congo red or calcofluor white (Fig. 4 bottom panel). The *yl-hog1Δ* strain displayed significantly increased resistance to both of these cell wall damaging agents compared to the wild-type MK1 strain, whereas *yl-HOG1* cells were more sensitive to Congo red and, to a lesser extent, calcofluor white, than MK1 cells. Thus, the increased resistance to cell wall damaging agents upon Hog1 loss is conserved in *Y. lipolytica*

### Activation of *yl-Hog1*

To investigate activation of yl-Hog1, western blotting was performed using an antibody that detects the phosphorylated, active, form of the kinase. As high osmolarity was the only stress that significantly inhibited the growth of *yl-hog1Δ* cells (Figs 2 and 3), yl-Hog1 activation was examined following exposure of cells to 1 M NaCl for 0, 10 and 20 minutes.

**Figure 5:**
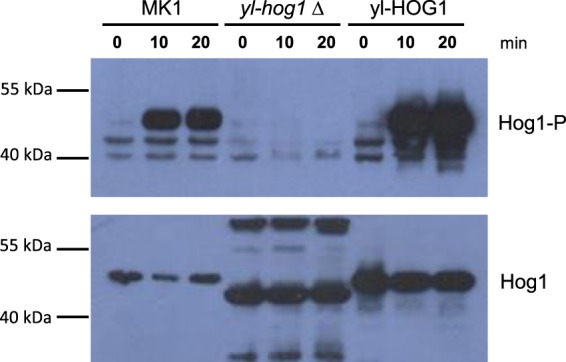
Western blot analysis of whole cell extracts isolated from *Y. lipolytica* wild type (MK1), *yl-hog1∆* and yl-HOG1 cells after treatment with 1 M NaCl for the specified times. The active form of protein (Hog1-P) was detected using an anti-phospho p38 antibody (upper panel). Total levels of yl-Hog1 protein were determined by probing the blot with an anti-Hog1 antibody (lower panel). The full-length blots are presented in Supplementary Fig. 1.

Phosphorylated Hog1 was clearly detected in protein extracts from both MK1 and *yl-HOG1* strains as a band running at the predicted size of 44.5 kDa (Fig. 5 upper panel). This was massively induced following NaCl treatment and, importantly, it was not present in any samples from the *yl-hog1∆* deletion strain. Moreover, the overexpression of yl-Hog1 in the strain *yl-HOG1* resulted in higher amounts of phosphorylated Hog1 both before and after NaCl stress compared to the wild-type MK1 strain. These results indicate that the detected protein was phosphorylated yl-Hog1, which is activated by osmotic stress.

The blot was stripped and reprobed for total Hog1 using a commercially available antibody raised against *S. cerevisiae* Hog1. As predicted, a band corresponding to yl-Hog1 was detected in samples from both wild-type MK1 and the overexpression yl-HOG1 strains before and after osmotic shock. Moreover, yl-Hog1 expression was significantly higher in yl-HOG1 cells compared to the wild type MK1 strain. A band of the predicted size was absent in samples from *yl-hog1Δ* cells, but there was non-specific binding to other proteins. The appearance of such cross-reactive bands, which are not present in MK1 cell extracts, indicates that deletion of yl-hog1 causes a change in the *Y. lipolytica* proteome.

### *yl-hog1*Δ cells are hyper-filamentous

**Figure 6:**
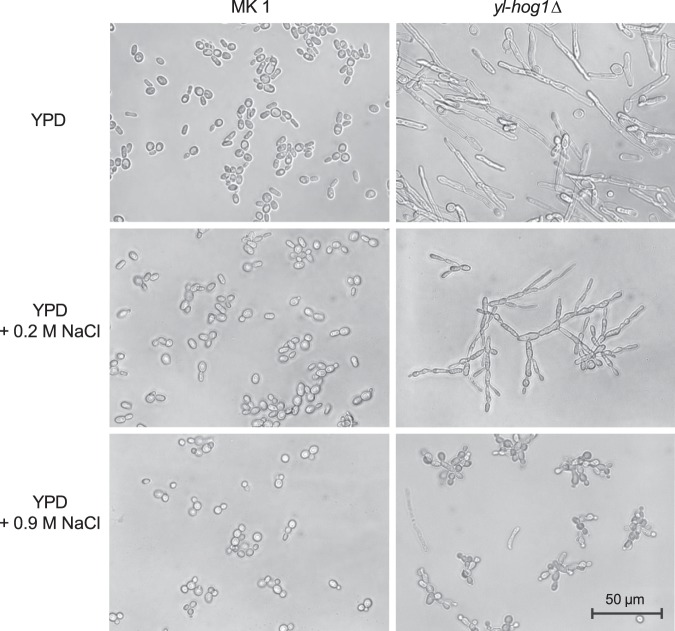
Morphology of *Y. lipolytica* strains MK1 and *yl-hog1Δ* after 24 h of growth in YPD medium supplemented with the indicated amounts of NaCl.

*Y. lipolytica* is a dimorphic yeast, and a number of conditions that cause transition from a budding ovoid form to a filamentous hyphal structure have been identified^31^. However, in YPD media at 30 °C with good aeration (shake flask experiments), the yeast-like form predominates, as shown for wild-type MK1 cells (Fig. 6). Strikingly, however, *yl-hog1∆* cells are largely filamentous under the same growth conditions (Fig. 6). This possibly underlies the ruffled colony morphology observed previously with *yl-hog1Δ* cells (Fig. 2). Thus yl-Hog1 seemingly functions to repress the morphogenetic transition under non-filament inducing conditions.

We next examined the impact of osmotic stress on the morphology of wild-type MK1 and *yl-hog1Δ* cells. Increased osmotic stress did not affect the morphology of MK1 cells, which continued to grow in the yeast form. However, exposure of *yl-hog1Δ* cells to low levels of osmotic stress (0.2 M NaCl) resulted in pseudohyphal like structures in contrast to the long, less branched, hyphal structures observed under non-stress conditions. At 0.9 M NaCl, the impact on the filamentous phenotype of *yl-hog1∆* cells was smaller, as the cells were much more ovoid. However, impaired cytokinesis was evident as chains of cells were prominent. These results indicate that yl-Hog1 plays an important role in regulating the dimorphic transition in *Y. lipolytica*.

### Erythritol production

Erythritol production is stimulated under high osmotic pressure. Therefore, as we have found that yl-Hog1 plays a central role in *Y. lipolytica* osmotic stress responses, we examined the importance of *yl-Hog1* in erythritol production. It should be noted that during erythritol production, yeast is subjected to a combination of stresses. The medium is minimal; apart from low concentrations (0.1%) of yeast extract, the compounds comprise only glycerol as a carbon source and inorganic salts. The presence of glycerol (100 g/L), which is the substrate for erythritol production, contributes to the high osmotic pressure, which is additionally increased by 26 g/L (450 mM) NaCl. Moreover, the pH is maintained at 3 during the process.

**Figure 7:**
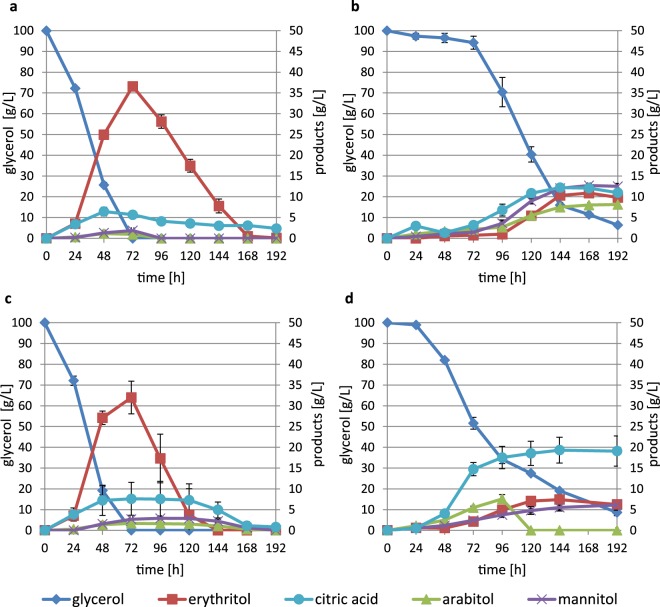
Production of erythritol by *Y. lipolytica* strains (**a**) MK1 (**b**) *yl-hog1∆* (**c**) yl-HOG1 (**d**) *yl-hog1∆* after 8 days of adaptive laboratory evolution. Experiments were performed in triplicate. Some error bars are too small to be visible on the chart.

*Y. lipolytica* MK1 completely depletes glycerol during 72 hours of growth (Fig. 7a). Over the same time period erythritol reaches the highest concentration (36.6 ± 1 g/L), but this is completely utilized before 168 h (Fig. 4a). The concentrations of citric acid, mannitol and arabitol were also measured, as these are by-products of erythritol production of the MK1 strain^20^. Citric acid reached the concentration of 6.4 ± 1 g/L at 48 h, while the concentration of mannitol and arabitol did not exceed 2 g/L.

Initially, the strain *yl-hog1∆* failed to grow in erythritol production medium (Fig. 7b). For the first 72 h nearly no yeast biomass was observed when collecting the samples for HPLC. The amount of glycerol also remained virtually unchanged. However, after a long lag phase, *yl-hog1∆* cells started to utilize glycerol and biomass appeared in collected samples. Erythritol was produced, but at much lower concentrations. Interestingly, similar concentrations of erythritol (9.8 ± 0.9 g/L), arabitol (8.15 ± 0.7 g/L), mannitol (12.5 ± 0.1 g/L), and citric acid (11 ± 0.9 g/L) were obtained. The long lag phase followed by sudden assimilation of glycerol suggested that the *yl-hog1∆* cells adapted to the growth conditions. This is similar to observations of *yl-hog1∆* cells grown in the presence of 0.9 M NaCl (Fig. 3d). In order to determine whether this adaptive response could be maintained, following the 8-day fermentation the *yl-hog1∆* cells were plated onto YPD agar plates for 48 h before re-inoculating into erythritol production medium (Fig. 7d). Although glycerol utilization was still slow, the lag phase was significantly shorter and lasted only 24 h. However, the highest erythritol concentration produced was not higher than in the first experiment (9.4 ± 0.5 g/L), although citric acid was produced in a relatively high amount (19.3 ± 3 g/L). The repetitive cultivation of microorganisms in initially unfavourable conditions in order to increase their fitness is known as adaptive laboratory evolution (ALE). The observed phenotype was not the result of contamination of the wild strain, which was validated by a PCR test for presence of the *YALI0E25135g* gene.

Overexpression of *yl-HOG1* also affected erythritol production (Fig. 7c). The glycerol assimilation rate was comparable with MK1, but the maximal concentration of erythritol produced was slightly lower (32 ± 4 g/L). However, the most interesting feature of the yl-HOG1 strain was very fast subsequent utilization of erythritol, as it was completely depleted 24 h earlier than in MK1.

## Discussion

The aim of the study was to establish the MAPK kinase crucial for responses to osmotic stress in the yeast *Y. lipolytica*. BLAST analysis identified the gene *YALI0E25135g* encoding the yl-Hog1 homologue of *S. cerevisiae* Hog1, the core element of the osmotic stress response HOG pathway. Deletion and overexpression of yl-Hog1 was performed and modified *Y. lipolytica* strains were subjected to extensive phenotypic and biochemical analyses.

yl-Hog1 kinase undoubtedly plays a key role in the response to hyperosmotic shock in *Y. lipolytica*. The *yl-hog1Δ* strain is sensitive compared to wild-type cells to NaCl or sorbitol, as observed in spot tests, Bioscreen C, and shake-flask experiments. The initial lack of growth might have resulted from impaired cytokinesis of *yl-hog1Δ* cells in presence of NaCl. However, after prolonged incubation in media with high osmolarity (YPD + 0.9 M NaCl or erythritol production medium), cellular growth resumed. A possible explanation of those results could be that some elements of the cellular response to hyperosmotic shock of *Y. lipolytica* are activated independently of yl-Hog1, or that whilst the immediate to mid-term responses to osmotic stress are Hog1 dependent, the longer term adaptive responses are Hog1-independent. Examples of such a mechanism are known in *S. cerevisiae*, where the membrane channels responsible for retention of osmolytes in the cell might be regulated by the TORC2 complex^32,33^. Another possibility is that the adaptation to hyperosmotic stress arises due to mutations generated during the long lag phase. The improved fitness of *yl-hog1Δ* cells on repeated exposure to erythritol production medium supports this hypothesis. This could be considered as an example of adaptive laboratory evolution (ALE), which is a method frequently used in metabolic engineering^34^. ALE could be considered in further studies on the HOG pathway, as sequencing of osmo-adapted *yl-hog1∆* mutants might help to identify other elements of the cellular response to osmotic stress. However, first more research is needed to investigate the molecular basis for this and whether the observed adaptation is a result of epigenetic changes or gene mutations.

The involvement of Hog1 in the response to temperature shock varies depending on the yeast species – it participates in both cold and hot stress responses in *S. cerevisiae*^35,36^, but in neither of them in *C. albicans*^14^. The results suggest more similarity to *C. albicans*, as deletion of yl-Hog1 did not have any negative impact on growth even during incubation at 4 °C.

Incubation at temperatures above the 28–30 °C optimum was more harmful for *Y. lipolytica*. The overexpression *yl-HOG1* strain had the highest proportion of surviving colonies at 35 °C, which might suggest that yl-Hog1 plays a role in the heat shock response. However, the evaluation of its influence will require further research and optimization of culture conditions.

Oxidative stress is known to be an additional activator of the HOG pathway in *C. albicans*^37^, *S.-pombe*^38^, and to a certain extent also in *S. cerevisiae*^39^. Mutant *yl-hog1∆* cells were not more sensitive either to H_2_O_2_ or menadione, indicating that yl-Hog1 is not essential for responses to oxidative stress triggered by these agents. On the other hand, the strain *yl-HOG1* was more resistant to menadione. This is particularly interesting, considering that overexpression of yl-Hog1 did not promote resistance to osmotic stress or H_2_O_2_-mediated oxidative stress. The cellular response to oxidative stress in yeast is very complex, involving a suite of signalling pathways^40^; thus it may be difficult to tease out the mechanism underlying menadione resistance in cells overexpressing yl-Hog1. Differences between responses to H_2_O_2_ and menadione are, however, not unique for *Y. lipolytica*, as proteomic analysis of *C. albicans* revealed variations in the expression of some cell wall proteins in response to different oxidative agents^41^.

The most characteristic feature of the strain *yl-hog1Δ* is the filamentous growth in conditions where the yeast form should prevail. *Y. lipolytica* can undergo dimorphic transition. Factors that are known to induce filamentous growth are: reduced availability of dissolved oxygen^31^, hydrophobic carbon sources^42^ and heavy metal stress^43^. None of these factors were applied in this study, and the MK1 strain remained in the yeast-like form. Similar observations of increased filamentous growth of *hogΔ* mutants of *C. albicans*^44^ and *S. cerevisiae* were reported^45^. In both cases it was recognized that Hog1 negatively regulates transcriptional factors that induce filamentous growth^46,47^. Such mechanisms are necessary to prevent inappropriate activation of other MAPK cascades, which could occur because some elements are incorporated in different cascades. In *S. cerevisiae* the MAPKKK Ste11 is involved not only in the HOG pathway, but in MAPK pathways regulating cell wall integrity, the mating response and dimorphism^12^. Ste11 is the only element of *Y. lipolytica* MAPK pathways that has been characterized so far, and it was described as important for mating and switching to the filamentous form^48^. It is still not known whether Ste11 relays osmotic stress signals in *Y. lipolytica* or, like in *C. albicans*, is dispensable, as Ssk2 is the sole MAPKKK regulating Hog1^16^.

Regardless of whether the HOG and filamentous growth cascades share Ste11 MAPKKK kinase, the role of Hog1 kinase to prevent spurious activation of other MAPKs is conserved between different yeast species. This is indicated by the effect of yl-Hog1 deletion on cell wall structure. The strain *yl-hog1Δ* has significantly increased resistance to the cell wall damaging agents Congo red and calcofluor white. Similar phenotypes are observed in *S. cerevisiae*^29^ and *C. albicans hog1Δ* mutants^30^. In *C. albicans* it was found that this was due to inappropriate activation of Cek1, which is the MAPK kinase responsible for cell wall biogenesis^49^.

The deletion of yl-Hog1 also has a negative impact on cytokinesis, which it is revealed after a small increase in osmotic pressure. In medium with 1% NaCl the strain *yl-hog1Δ* was unable to complete cytokinesis and cells remained connected. Similar observations were made in *C. albicans* by Alonso-Monge *et al*.^30^, who examined *hog1Δ* mutants using scanning and transmission electron microscopy, which revealed that the septum between cells was completed, but that they were still connected by the outer layer of the cell wall.

The effects of yl-hog1 deletion on filamentous growth, impairment of cytokinesis and increased resistance to cell wall perturbing agents illustrate that the role of this MAPK extends beyond the regulation of stress responses. These results indicate the existence of interactions between HOG and other signalling pathways, which are conserved in a number of fungi, but have not been identified in *Y. lipolytica* yet. However, despite similarities between the *Y. lipolytica* Hog1 pathway and those in *S. cerevisiae* and *C. albicans*, the downstream response to the osmotic stress is very distinct. *Y. lipolytica* produces the polyol erythritol instead of glycerol, and even uses glycerol as a substrate for this process.

So far, the only studies of Hog1 in erythritol-producing yeasts were performed by Li *et al*. on *Trichosporonoides oedocephalis*^50^. *T. oedocephalis* simultaneously produces both erythritol and glycerol from glucose. The to-Hog1 deletion caused a significant reduction of glycerol and increase in erythritol production. These results suggested that erythritol production might be controlled by pathways other than HOG, or is negatively regulated by HOG. Thus at the early stages of our work we speculated that impairment of HOG might help reveal a similar but as yet unknown pathway regulating the hyperosmotic stress response in *Y. lipolytica*. However, the impact of yl-Hog1 deletion was so devastating for growth and erythritol production that this hypothesis was excluded. In *Y. lipolytica* there is not a mechanism that could rapidly substitute for HOG in the short-term response to osmotic shock.

The fact that *T. oedocephalis hog1Δ* still can efficiently produce erythritol while *Y. lipolytica* cannot might have resulted from the use of different media in the experiments. In the case of *T. oedocephalis*, osmotic stress was induced only by a high concentration of glucose (200 g/L). In contrast, the medium optimized for erythritol production for Y*. lipolytica* contains 100 g/L glycerol and 26 g/L NaCl, and thus imposes a higher osmotic pressure. Thus there is a possibility that yl-Hog1 is not needed directly for the induction of erythritol production, but under the growth conditions used for its synthesis it is necessary for *Y. lipolytica* to remain metabolically active. Investigations into the downstream responses of yl-Hog1 are needed to determine whether this pathway plays a direct or indirect role in erythritol production.

## Conclusion

yl-Hog1 kinase is essential for rapid responses to hyperosmotic stress in *Y. lipolytica*, and has additional roles in regulating cell wall integrity and the yeast-to-hyphae transition. The *yl-hog1Δ* mutant was unable to produce high amounts of erythritol, and overexpression of yl-Hog1 also impaired erythritol production parameters. Thus yl-Hog1 regulates many cellular processes and is likely to be part of a complicated network of signalling pathways.

## Electronic supplementary material

Supplementary information
