# Supplementary material for: Influence of ylHog1 MAPK kinase on Yarrowia lipolytica stress response and erythritol production
Source: Sci Rep. 2018 Oct 3;8:14735. doi: 10.1038/s41598-018-33168-6 (PMC6170386; doi:10.1038/s41598-018-33168-6)
Supplement: Supplementary file 1 — Supplementary information [file 41598_2018_33168_MOESM1_ESM.pdf]

Influence of ylHog1 MAPK kinase on *Yarrowia lipolytica* stress response and erythritol production – Supplementary Information

Dorota A. Rzechonek[1], Alison M. Day[2], Janet Quinn[2], Aleksandra M. Mirończuk[1*]

1) Wrocław University of Environmental and Life Sciences, Department of Biotechnology and Food Microbiology, Chełmońskiego 37, 51-630 Wrocław, Poland
2) Newcastle University, Institute for Cell and Molecular Biosciences, Framlington Place Newcastle upon Tyne NE2 4HH, United Kingdom

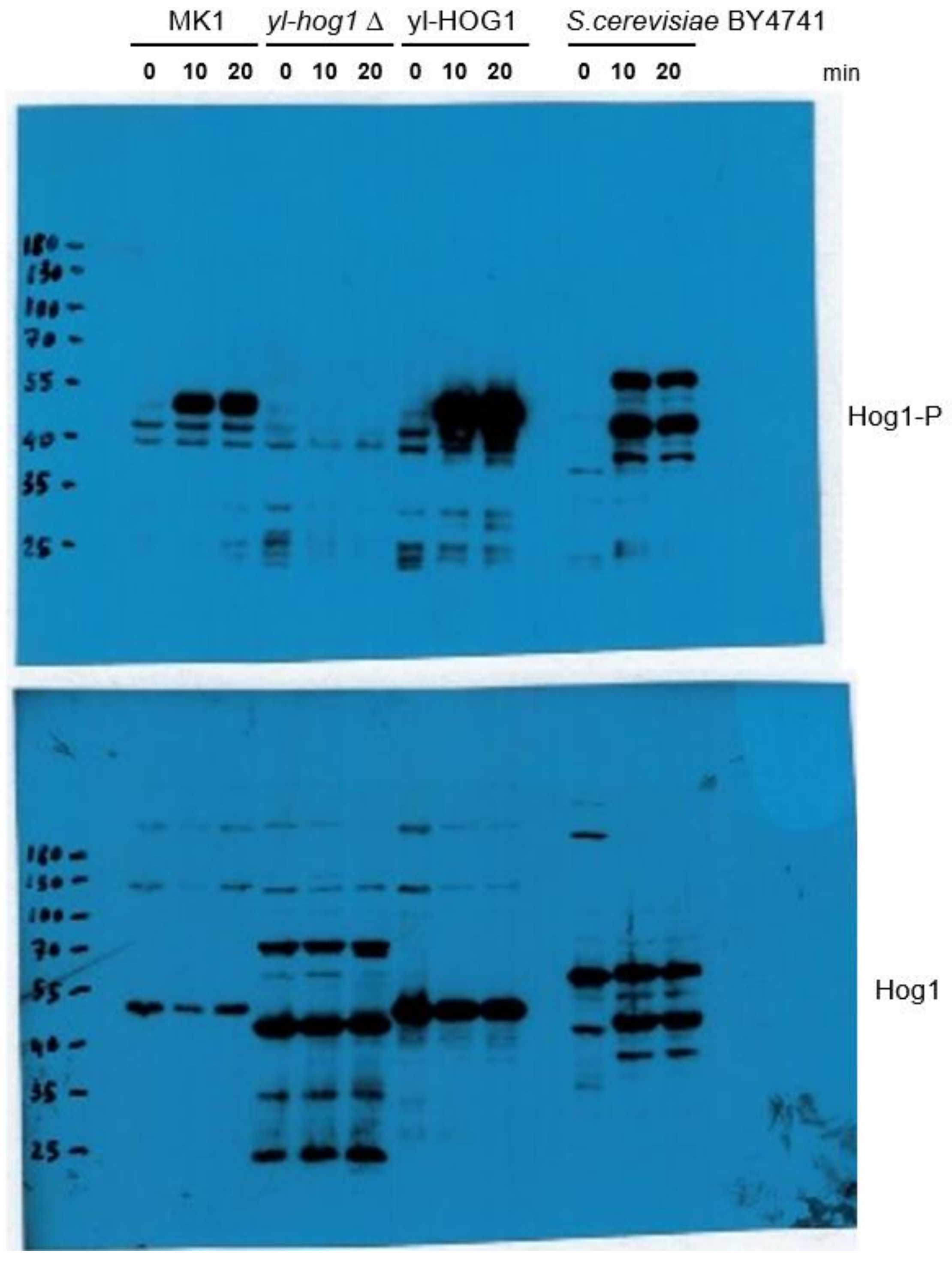

Fig 1 Western-blot analysis of whole cell extracts isolated from *Y. lipolytica* wild type (MK1), yl-hog1Δ and yl-HOG1 cells after treatment with 1M NaCl for the specified times. The active form of protein (Hog1-P) was detected using an anti-phospho p38 antibody (upper panel).

Total levels of yl-Hog1 protein were determined by probing the blot with an anti-Hog1 antibody (lower panel). *S. cerevisiae* BY4741 was used as a positive control and was processed parallel to *Y. lipolityca* samples. *S. cerevisiae* Hog1 is bigger than *Y. lipolytica* homologue and is visible as the upper band. Unspecific lower band might be a result of optimisation of protocol for *Y. lipolytica.*

PageRuler™ Prestained Protein Ladder, 10 to 180 kDa was used as a marker. The bands of marker were visible on membrane blot, and copied manually on the photographic film.
